# Do changes in snow conditions have an impact on snowmaking investments in French Alps ski resorts?

**DOI:** 10.1007/s00484-020-01933-w

**Published:** 2020-05-28

**Authors:** Lucas Berard-Chenu, Jonathan Cognard, Hugues François, Samuel Morin, Emmanuelle George

**Affiliations:** 1 Univ. Grenoble Alpes, INRAE, LESSEM, 38000 Grenoble, France; 2 Univ. Grenoble Alpes, Université de Toulouse, Météo-France Grenoble, CNRS, CNRM, Centre d’Etudes de la Neige, 38000 Grenoble, France

**Keywords:** Snowmaking investment, Snow conditions, Ski resorts, French Alps

## Abstract

This study investigates the relationship between snow conditions and snowmaking investments for over 100 French Alps ski resorts. Investment trends represent a critical issue in capital-intensive sectors such as the ski industry. The data are based on snow reliability indicators and snowmaking investments covering 1997–2014. Descriptive statistics reveal that snowmaking has been the second investment item for ski resorts regardless of the elevation or ski resort size. The study finds that snowmaking investments are highly negatively correlated to snow conditions of the prior year for small and medium-sized ski resorts. Other factors are also likely to play a significant role in driving snowmaking investment dynamics.

## Introduction

Winter tourism is an important industry in the European Alps. France ranks within the two top ski tourism destinations in Europe, depending on the year, with annually about 55 million skier visits (DSF 2017; Vanat 2019). The French Alps represent more than 80% of total annual French skier visits. In the French Alps, the share of tourism employment is almost 8% and tourism expenditures related to ski resorts amount to about 6.5 billion EUR (Atout France 2012). In Austria, where the ski industry is also a key sector, winter sports generate almost 7.4 billion EUR in direct value added every year (Arbesser et al. 2010).

The ski tourism industry is highly sensitive to meteorological and snow conditions, in particular their interannual variability. Poor snow seasons have direct impacts on the tourism industry. Snow scarcity affects ski resort operators but also all surrounding stakeholders (e.g. host providers, restaurants, merchants, ski instructors, rental and sale shops of sports equipment, transport operators) connected to the ski industry. In addition to the interannual variability, past and future climate simulations for the twenty-first century indicate an ongoing reduction of snow amounts, especially in low to mid-elevation up to around 1500 m above sea level, superimposing on persistent interannual variability (Beniston et al. 2018; Verfaillie et al. 2018; Spandre et al. 2019a, 2019b). Hock et al. (2019) indicate that climate change has a negative impact on the tourism sector in the Alps, especially for the operating conditions in the winter season, with high financial risks for communities that rely on tourism income.

For several decades, the French ski industry has undergone deep changes, such as shifts in demand and consumer preferences, the fact that ski tourism is a mature market with a higher competition between resorts, changing governance contexts, and upgrading of tourism supply offer, in addition to emerging climate change impacts (Cuvelier 1997; Gerbaux and Marcelpoil 2006; Tuppen 2000). One of the key changes that have affected this industry over the past decades is the inception and development of snowmaking (Spandre et al. 2015).

Snowmaking plays a routine role in the ski industry, where it contributes to snow management in advance and during the season. Snowmaking has only recently been fully integrated into scientific assessments of ski resort exposure and vulnerability under climate change (Steiger et al. 2019; Abegg et al. 2020; Hoegh-Guldberg et al. 2018). Although snowmaking is increasingly taken into account in climate change impact studies (Spandre et al. 2019a; Spandre 2016), far less studies have analyzed the motivation and the development strategy for snowmaking equipment, which are still poorly understood, with implications for the assessment of the impact of snowmaking on the operating and business model of the ski tourism industry.

There is a broad consensus on the fact that a series of poor snow seasons at the end of the 1980s and at the beginning of the 1990s (Durand et al. 2009) led to the inception of snowmaking use in the French Alps (Gauchon 2009; Paccard 2010). Since snow conditions have triggered the onset of use of snowmaking, it is appropriate to assess whether there is a relationship between snow conditions and snowmaking development over the past decades. Several previous studies show the increasing trend for the use of snowmaking in France (Paccard 2010; Badré et al. 2009; Spandre et al. 2015). The purpose of the present study is to assess to what extent changes and fluctuations in snow conditions can explain variations in the general trend for snowmaking investments in French Alps ski resorts. The study is also an opportunity to contribute to a better situational analysis of snowmaking investments, complementing the study of Falk and Vanat (2016) who found that investment in snowmaking is widespread and cumulated past investment has a positive impact on the number of skier visits. To do so, we provide a situational analysis of snowmaking activities in the French Alps, with a focus on overall investments in ski resorts. In order to reach its goal, our study is based on econometric analysis of snowmaking investment and snow reliability panel data sets.

The paper is structured as follows. “Conceptual background and development of assumptions” provides an overview of the existing literature in the field of the links between ski tourism and snowmaking. “Materials and methods” introduces the data and the methodology. “Results” provides results, in the form of descriptive statistics and the empirical results drawn from our modeling. “Discussion” discusses the results and the empirical model and gives concluding remarks.

## Conceptual background and development of assumptions

Knowledge about snowmaking is mainly found in the climate change and ski tourism vulnerability literature. In the 2000s, climate change studies about ski tourism started to take snowmaking into account. Reference studies (Bürki et al. 2005; Elsasser and Messerli 2001) and an OECD report (Abegg et al. 2007) presented what has become a general agreement: snowmaking is “the most widespread adaptation strategy used by ski area operators” (Abegg al. 2007)*.* Several studies highlighted the key role of snowmaking systems in shaping the snow reliability of mountain ski resorts (Scott et al. 2003; Scott and McBoyle 2006; Steiger and Mayer 2008; Gonseth 2013; Pons et al. 2015). Henceforth a major issue was to realistically account for snowmaking in ski resort vulnerability assessment studies. Gradually, snow reliability models as well as ski tourism vulnerability assessments take into account snowmaking (Scott et al. 2003; Steiger 2010; Dawson and Scott 2013; Pons et al. 2015; Pons-Pons et al. 2012; Steiger et al. 2019; Steiger and Stötter 2013; Spandre et al. 2019a, 2019b; Abegg et al. 2020). Whether snowmaking remains a relevant adaptation strategy under future climate change has become a growing concern in the literature and triggers intense public debate in mountainous regions: technical feasibility under a warmer climate, concerns about water and energy resource requirements and their financial implications have increasingly been discussed (Steiger et al. 2019). In addition to environmental debates, because the use of snowmaking requires high investment and operating costs, snowmaking development and its socio-economic implications have become a central question. Gonseth (2008) showed that the positive impact of snowmaking on the Swiss ski resorts’ EBITDA (earnings before interest, tax, depreciation, and amortization) decreases as the level of snowmaking investments increases. When a threshold of 30 km of ski slopes covered with snowmaking systems is exceeded, additional snowmaking has a negative impact on the ski resort’s EBITDA. Analyzing a snowmaking investment dataset, Falk and Vanat (2016) estimated that above 6.5 million EUR invested, cumulated snowmaking investment does not lead to higher skier visits in French ski resorts. Damm et al. (2014) performed a cost revenue analysis and predicted a future price increase in ski lift tickets in a ski area in Austria due to expected rising snowmaking operating costs. In an econometric study of corporate adaptation to climate change, Hoffmann et al. (2009) indicated that snowmaking extension is one adaptation measure among many for ski lift operators. In a vulnerability assessment of ski tourism in Germany and Turkey, Demiroğlu (2016) stated that snowmaking adaptation strategy can lead resorts on a path dependency, with challenging fixed and operational expenses. The way snowmaking is considered in most of the studies suggests that snowmaking is exclusively an adaptation measure to increase the snow reliability under climate variability and climate change, with some adaptation costs to assess.

However, among the growing amount of climate change perception studies on the tourism supply side (Abegg et al. 2008; Luthe 2009; Scott et al. 2012; Trawöger 2014), several factors about ski lift operators behavior indicate that snowmaking is not only implemented to face snow variability and climate change. Trawöger (2014) has conducted interviews with ski tourism stakeholders in the Austrian Alps and her results showed that ski lift operators were convinced that changing climatic conditions will affect the ski industry in the mid- and long-term. These observations are compatible with findings about climate change vulnerability perception from other business sectors. For instance, Arnell and Delaney (2006) showed that water supply companies in England consider climate change impacts as one threat among others. Scott et al. (2012) stated that a difference exists between business and climate change timelines and highlighted that short-term planning horizon prevails among ski industry stakeholder decisions. Because of their relatively short (a few decades) depreciation period, capital investment decisions require only a mid-term planning. In view of this dissonant timelines, snowmaking investments are therefore probably carried out to meet current rather than future needs of ski lift operators.

To the best of our knowledge, only a few studies have addressed ski lift operator motivation to invest in snowmaking. Steiger and Mayer (2008) and Spandre et al. (2016a) have pointed out that some ski resorts invest in snowmaking even in high elevation areas. Although the highest ski resorts might have a snow-depth minimum threshold higher than the 30 cm generally considered, these authors’ findings illustrate that the decline of snow conditions, regardless of the climate change scenarios, is not a sufficient reason to explain the rise of snowmaking at high elevation. In addition to climate change, Steiger and Mayer (2008) identified four conditions that raise snowmaking use: variability of precipitation in pre and early winter, competitive economic pressure, global trend in tourism, and specific trends in ski tourism. Based on a document from the professional association of the French cable car operators (SNTF 2002), Paccard (2010) also reported that in France, snowmaking results from different motivations. Though the specification lightly differs, snowmaking motivations mentioned by Paccard (2010) are congruent with those laid out by Steiger and Mayer (2008): to provide a base layer snowmaking to secure the scheduled openings of the ski resort, to guarantee the staging of international ski competitions, or to ensure the operation of the most strategic ski lifts. It remains unclear how snowmaking development is managed by ski lift operators, perhaps because even themselves take an ambiguous position on snowmaking. Wilson et al. (2018) indicated that snowmaking technical use has evolved: ski resorts have increased their capacities to produce a larger amount of snow within a shorter period. Campos Rodrigues et al. (2018) mentioned technical progress from snowmaking system suppliers, now snowmaking systems can produce snow until − 1.5 °C compare to figure of − 4 °C in the 1990s. Based on current knowledge, snowmaking development meets various purposes: counteracting the declining snow reliability in low-elevation areas, providing a snow guarantee for customers, assuring the best snow conditions on the ski slope, and preserving competitiveness within a mature European ski tourism market (Spandre et al. 2016a). Snowmaking can thus be considered as a coverage that makes possible the ski area exploitation for ski lift operators, more sustainable than financial hedges (Tang and Jang 2011) and that can be complemented with snow farming process, i.e., storage of snow from one season to the next (Grünewald et al. 2018). Several articles have described the past development of snowmaking in the French Alps in a descriptive manner (e.g., Spandre et al. 2015; Spandre 2016), but without analyzing the decision process leading to investments. The current study addresses specifically the relationships between ski resort snowmaking investment and snow reliability indicators. Firstly, it contrasts snowmaking investments with other ski resort investment trends. Secondly, it characterizes the relationship between snowmaking investment figures and snow conditions.

To address our research issue, the following assumptions are made to set a framework. Firstly, we analyze the influence of ski resort size on investments. We assume that snowmaking investment response could be different depending on the ski resort size. Large ski resorts are more often higher than small ski resorts, with a higher number of snow reliable days. They operate high-performance ski lifts, faster and more comfortable than in smaller ski resorts. They attract more visitors, thus generate a bigger business volume. Because of their high turnover, the largest ski resorts are able to have steady investment programs. The largest ski resorts invest large amounts in snowmaking facilities (Falk and Vanat 2016) and they might also invest more frequently than smaller resorts.

Secondly, we test the time lag relevance between snow reliability indicators and snowmaking investments. Apart from a widely held opinion that snowmaking use soared in the alpine region after a number of poor snow seasons at the end of the 1980s and the beginning of the 1990s, there is no literature on such relationships. It is unlikely that a direct relationship could exist between a meteorological variable and its economic potential consequences for ski lift operators’ investments. A time lag likely exists, because a ski lift operator cannot react to a poor snow season by new snowmaking facilities within the same year. This assumption is consistent with Falk and Steiger (2018), who assumed that warm seasons could lead to increased investments into snowmaking facilities in subsequent years. Thus, if a relationship exists between these two variables, it is likely that there is a time lag between the two with a negative correlation, i.e., *a reduction in snow reliability would increase upcoming snowmaking investments.*

## Materials and methods

### Ski resorts characteristics from the BD Stations database

We characterized the main geographical and technical features of each considered ski resort using the BD Stations (Marcelpoil et al. 2012; François et al. 2014). BD Stations is a comprehensive database of French Alps ski resorts. It includes information on ski resorts’ ski lift power, which is defined as the sum of the ski lift power of all ski lifts in a given ski resort. The ski lift power of a ski lift is defined and computed as the product of the elevation difference between the top and bottom of a ski lift and its capacity, i.e., the number of persons that can be carried per hour. It is a better indicator to distinguish ski resort diversity than the number of ski lifts or the total number of slopes and is widely used in various studies about French ski resorts (Goncalves 2013; François et al. 2014; Spandre et al. 2015, 2016a, 2019a, 2019b). It was used to split our initial sample between four categories (see Table 1).

**Table 1:** Ski resort categories by ski lift power

Resort category*	Small resort (S)	Medium resorts (M)	Large resorts (L)	Very large resorts (XL)
Ski lift power (SLP) unit: km.pers./h	SLP < 2500	2500 < SLP < 5000	5000 < SLP < 15,000	15,000 < SLP

*Domaines Skiables de France (*DSF* formerly known as *SNTF*, the professional association of the French cable car operators)

### Investment data set

Investment figures provide valuable information on the behavior of ski lift operators. Few other indicators can be assessed to understand ski tourism from the supply side. Ski ticket price (Wolff 2014), investments and turnover, or, best of all, benefits (Gonseth 2008) are useful proxies to assess the strategic economic behavior of a ski resort. Falk (2009) and Falk and Tveteraas (2019) showed that ski lift operators act as a high capital–intensive industry as cable car equipment purchase requires substantial investments. The ski tourism industry is also a capital-intensive business for public local communities. Uhaldeborde (2007) assessed that a ski tourism-oriented economy doubles the equipment investment rate of local authorities. French mountainous areas reach 55% of national tourism investment, although they represent only 15% of the turnover (Atout France 2012). Several methods exist to estimate the capital intensity ratio. We measure it as the product of total fixed assets (k€) divided by full-time equivalent employee (FTE). Employment data are provided by the French Central Agency for Social Security organizations (L’Agence centrale des organismes de sécurité sociale [ACOSS], website:https://www.acoss.fr/home/observatoire-economique/donnees-statistiques/bases-de-donnees.html) while total fixed assets have been extracted from private companies’ balance sheets provided by the Diane database (Source: Bureau van Dijk, website: https://www.bvdinfo.com/en-gb/our-products/data/national/diane).

Investment data used in this article originate from an analysis of the reporting of investments by the professional journal *Montagne Leaders.*^1^ This journal sends every year a survey to each French ski resort. Ski resorts fill the survey in a declarative manner. To be ranked as one of the “Top 100 French ski resorts” published by *Montagne Leaders* is also an evidence of renown for ski lift operators. The total amount of investment is distributed in different parts: snowmaking investments, investments in new ski lifts, or ski lift maintenance investments. Because the questionnaire has changed several times in 1994, 1996, 1999, 2000, and 2005, we focused our first analysis on the 2005–2016 period and selected five types of investment, which have always been considered. Although collected by a non-scientific and unofficial organization, these data hold significant value. The journal team has strengthened its methodology over time with expertise provided by *Atout France*, which is the national organization responsible for promoting France as a tourism destination.^2^ In its own publications about ski tourism, *Atout France* widely uses the *Montagne Leaders* investment data set (Atout France 2018, 2016, 2015, 2013). Initially, the methodology was not described, but since the beginnings of the 2000s, a quick summary explains the methodology and data panel. Since 2014, the questionnaire used for the survey is also available on the website of the journal. Falk and Vanat (2016) have already used this dataset and noticed missing values before 2005.

We have considered two time periods and corresponding sets of ski resorts. Collection A spans 131 ski resorts, for which data is available for the 5 types of investment (new ski lift, ski lift maintenance, snowmaking, ticketing, and ski slope remodeling) and cover the period from 2005 to 2016. Collection A contains ski resorts, which have at least invested in one of the five categories over this time period.

Collection B spans 100 ski resorts, and focuses on snowmaking investments only, from 1997 to 2014, and only contains ski resorts, which have invested at least once in snowmaking over this entire period of time. A thorough analysis of the archives of the journal was necessary to develop this unique dataset. In general, the average answer rate over the 1997–2014 period relating to the snowmaking survey is quite low: 44% (± 8). A more precise overview shed an additional light: the smaller the ski resort is, the lower its probability to answer the survey (See Table 2). The declining answer rate depending on resort size can have several explanations: the smallest resorts do not invest each year in snowmaking facilities because they have a discrete investment strategy. They might have difficulties to set an annual investment strategy. Investment information can also be harder to get by the smallest ski resorts: small staff, no dedicated person for investment controlling, etc.

**Table 2:** Average answer rate to *Montagne Leaders* snowmaking survey for ski resorts in the French Alps (1997–2014)

Ski resort size	S	M	L	XL	Total
Number of ski resorts in the BD Stations database	65	20	39	15	139
Answer rate (%)	17	47	73	90	44
	(± 7)	(± 17)	(± 10)	(± 10)	(± 8)

The datasets A and B include missing values. However, since the survey results are provided only for ski resorts with investment, we have assigned 0€ value to missing values.

### Snow reliability conditions

Snow conditions in each ski resort were computed using the SAFRAN-Crocus model chain (Durand et al. 2009), applied specifically for ski resorts, based on spatial and technical characteristics of ski resorts named gravitational envelopes (François et al. 2014, 2016) through the use of the Crocus-Resort snowpack model (Spandre et al. 2016b). This model chain was used to assess past and future changes in snow reliability in French Alps ski resorts (Spandre et al. 2019a; Spandre et al. 2019b). The snow reliability index (%) is defined as the fraction of the surface area of the gravitational envelope with a minimum quantity of snow for skiing. A surface is declared snow reliable when the snow mass exceeded 100 kg m^−2^, i.e., 20 cm of snow with a density of 500 kg m^−3^ (Spandre 2016; Spandre et al. 2019b). The modeling system provides indicators of the annual scale snow conditions, focusing on the Christmas and winter holiday periods, which are of critical importance for ski resorts economics (Spandre 2016; Spandre et al. 2019b). In this article, we assess snow conditions for each ski resort using simulations of natural snow conditions only (i.e., without grooming and snowmaking) and the corresponding reliability index.

### Empirical model

This study seeks to assess and qualify the relationship between snowmaking investments and snow reliability conditions. This is tested through an econometric analysis. To estimate the impact of the snow reliability index in the prior year on snowmaking investment trends in ski areas, we present an empirical model of the snowmaking investment function. In tourism literature, snowmaking investments are mostly considered an explanatory variable from the tourism demand function (Falk and Vanat 2016) rather than the dependent variable. Since no literature exists on snowmaking investment determinants, we set an empirical model as a function where output is snowmaking investments mainly determined by past natural snow reliability.

The linear model to be estimated is:

$$ {I}_{i,t}={\alpha}_i+{\beta}_1{Snow}_{i,t-1}+{\lambda}_t+{\varepsilon}_{i,t} $$

where *i* and *t* denote the ski area and the year. The left-hand variable *I*_*i,t*_ denotes the snowmaking investment, deflated by the GPD deflator. *Snow* is the natural snow reliability index. Only past snow reliability values (*t* − 1) are considered. *β*_1_ represents the respective coefficient. In line with our second assumption that we test, a negative sign is expected for β_1._ *α*_*i*_ is the ski resort specific effects, it captures all the time-invariant factors of each ski resort (e.g., ski resort governance model, be a part of a large company or the availability of water supply). *λ*_*t*_ is the time-specific effect; it captures factors that are common for all ski resorts, e.g., macroeconomic conditions. *ε*_*i,t*_ corresponds to the idiosyncratic disturbance.

We employ 4 widely used estimators for linear panel data: a pooled estimator computed using ordinary least squares (Pooled_OLS), fixed-effects estimators with first differences-transformation (FE_FD), and a within-transformation (FE_WITHIN), and also a random effect estimator using generalized least squares (RE_GLS). The pooled estimator assumes that *α*_i_ = *α* ∀ *i*, i.e., α_i_ is constant for all ski resorts. In our case, this estimator is probably not the most efficient, because of the intrinsic characteristics of ski resorts that influence the snowmaking investments. The central distinction between fixed and random effects is whether the unobserved individual effect *α*_i_ incorporates elements that are correlated with the regressors in the model. There is little justification in economics for treating the individual effects as uncorrelated with the regressors (Greene 2019, p.414). As the random effects specification requires a strong assumption, a fixed-effects estimator is generally preferred (Wooldridge 2016). In our case, it seems reasonable to consider that the natural snow reliability index is strictly exogenous. We assess our model with the four estimators presented. We use the Hausman specification test (Hausman 1978) to determine the preferred specification between within-transformation (FE_WITHIN) and the random effect estimator (RE_GLS). If the null hypothesis (H_0_) of no correlation is not violated, fixed-effects and random effects estimators are consistent, but fixed-effects specification is less efficient than the random effect. Under the alternative hypothesis (H_1_), a random effects estimator is inconsistent and biased, and fixed effects is preferred. We also check if the pooled estimator (Pooled_OLS) is not more appropriate than a random effects estimator with the Breusch-Pagan Lagrange Multiplier test (Breusch and Pagan 1980). For the estimations, we use R’s plm package (Croissant and Millo 2008). The within-transformation (FE_WITHIN) and the random effects specification (RE_GLS) control time-specific effects.

## Results

### Capital intensity ratio

The capital intensity ratio calculation highlights the key role played by investments in the ski tourism industry. We estimate the capital intensity ratio for 6 main sectors of the tourism industry, based on industry groups of the French industry standard classification system (APE code). Table 3 shows the mean capital intensity ratio by the tourism industry sector in France over the 2009–2016 period.

**Table 3:** Capital intensity ratio by the tourism industry sector in France (2009–2016)

	Tourist accommodation	Traditional catering	Casinos and gambling	Theme parks	Balneology and body care	Cable car transportation
Number of companies in the Diane database	18,995	36,281	280	418	1235	99
capital intensity ratio (k€/FTE employee)	63 ± 4	9 ± 1	40 ± 6	89 ± 24	28 ± 2	158 ± 20

Unsurprisingly, catering is not a capital intensive sector while cable car transportation is the most capital intensive sector (158 k€/FTE). Such a high level can be explained by the low number of full-time equivalent employee in this industry (around 9500) related to the seasonal activity in ski resorts. Ski lift operations involve high investments to maintain competitive facilities such as cable cars and gondolas. Hence, the ability to invest is a crucial issue for ski lift operators.

### Descriptive statistics

We provide descriptive statistics based on our 2 samples. Collection A (2005–2016) is only used for a descriptive and comparative purpose while collection B (1997–2014) is used in our econometric modeling.

Collection A (2005–2016) contains 131 ski resorts. It highlights the wide variety of French Alps ski resorts with few very large ski resorts and many smaller resorts. All M, L, and XL ski resorts from the BD Stations are included in the sample with only 8, out of 65, S (small) ski resorts missing.

Table 4 displays several descriptive statistics based on collection A. The ski resort size is linked to elevation; the larger resorts are generally located at higher elevation. Small ski resorts represent more than 40% of our sample while there are 11% very large ski resorts. However, very large resorts represent the major amount of investment: 44% while smaller resorts only account for about 5%. This snowmaking investment distribution is consistent with the ski lift power distribution, whereby very large and large ski resorts represent respectively 42% and 45% of the total ski lift power in the French Alps, while medium and small ski resorts count for 9% and 5%, respectively.

**Table 4:** Descriptive statistics about investments 2005–2016 (12 years) as a function of ski resort size

	S	M	L	XL	Total
Number of ski resorts in the collection/(number of ski resorts in the BD Stations database)	57/(65)	20/(20)	39/(39)	15/(15)	131/(139)
Ski lift mean elevation (masl) weighted by SLP	1471	1683	1830	2088	1682
Cumulated investment in new ski lift (k€)	67,793	141,406	666,617	657,246	1,533,062
Cumulated investment in ski lift maintenance (k€)	14,457	28,829	127,747	190,401	361,434
Cumulated investment in snowmaking (k€)	28,872	46,841	232,351	234,516	542,580
Cumulated investment in ticketing (k€)	2641	4529	27,472	20,002	54,644
Cumulated investment in slopes tracks (k€)	6400	13,909	66,547	78,329	165,185
Total investment (k€)	120,163	235,514	1,120,734	1,180,494	2,656,905

All investments are in current prices, i.e., we ignore adjustment for inflation

**Fig. 1:**
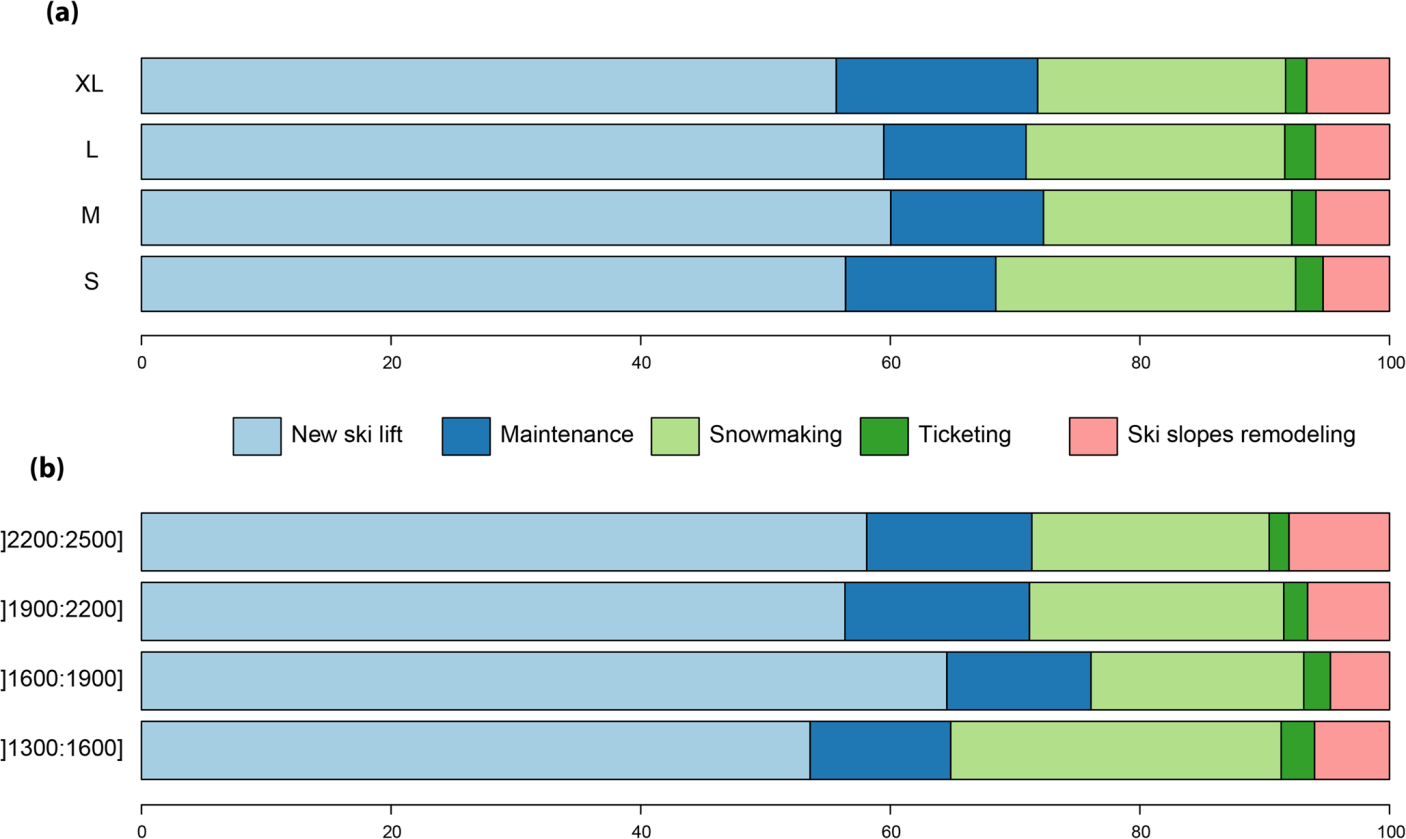
Distribution of investments over the 2005–2016 period, for the 5 main types of investment: new ski lift, ski lift maintenance, snowmaking, ticketing, and ski slope remodeling. Results are provided as a function of (**a**) ski resort size and (**b**) the mean elevation of the ski resort (by steps of 300 m)

Figure 1 shows the distribution of investments of ski resort operators over the period 2005–2016. Figure 1 (a) shows that new ski lifts, ski lift maintenance, and snowmaking together account for almost 90% of the ski lift operators’ investments. New ski lifts are, by far, the major investment item for ski lift operators. Snowmaking represents the second item of investment, which varies between 20 and 24% depending on the ski resort size. The share of snowmaking investments is rather stable regardless of the ski resort size.

According to Fig. 1(b), the investment distribution as a function of mean elevation follows almost the same pattern as Fig. 1(a). New ski lift investment remains the main cost item. However, some distinctions appear. Over the period studied, lower elevation ski resorts, with a mean elevation between 1300 and 1600 m, dedicated 26% of investments for snowmaking while the highest ski resorts allocated 19% of their investment to snowmaking. Ski resorts with a mean elevation between 1600 and 1900 have the lowest snowmaking investment rate (17%), preferring investments in new ski lifts. The lowest resorts have a higher allocation ratio for snowmaking. However, a decrease in snowmaking investment ratio according to the elevation does not clearly appear. The ski resorts at higher elevation are also concerned with snowmaking investment, consistent with Steiger and Mayer (2008).

We now turn to a longer time series collection B (1997–2014), specifically focusing on snowmaking investments and used in our modeling. Table 8 in the Appendix exhibits the list of ski resorts used in collection B.

**Fig. 2:**
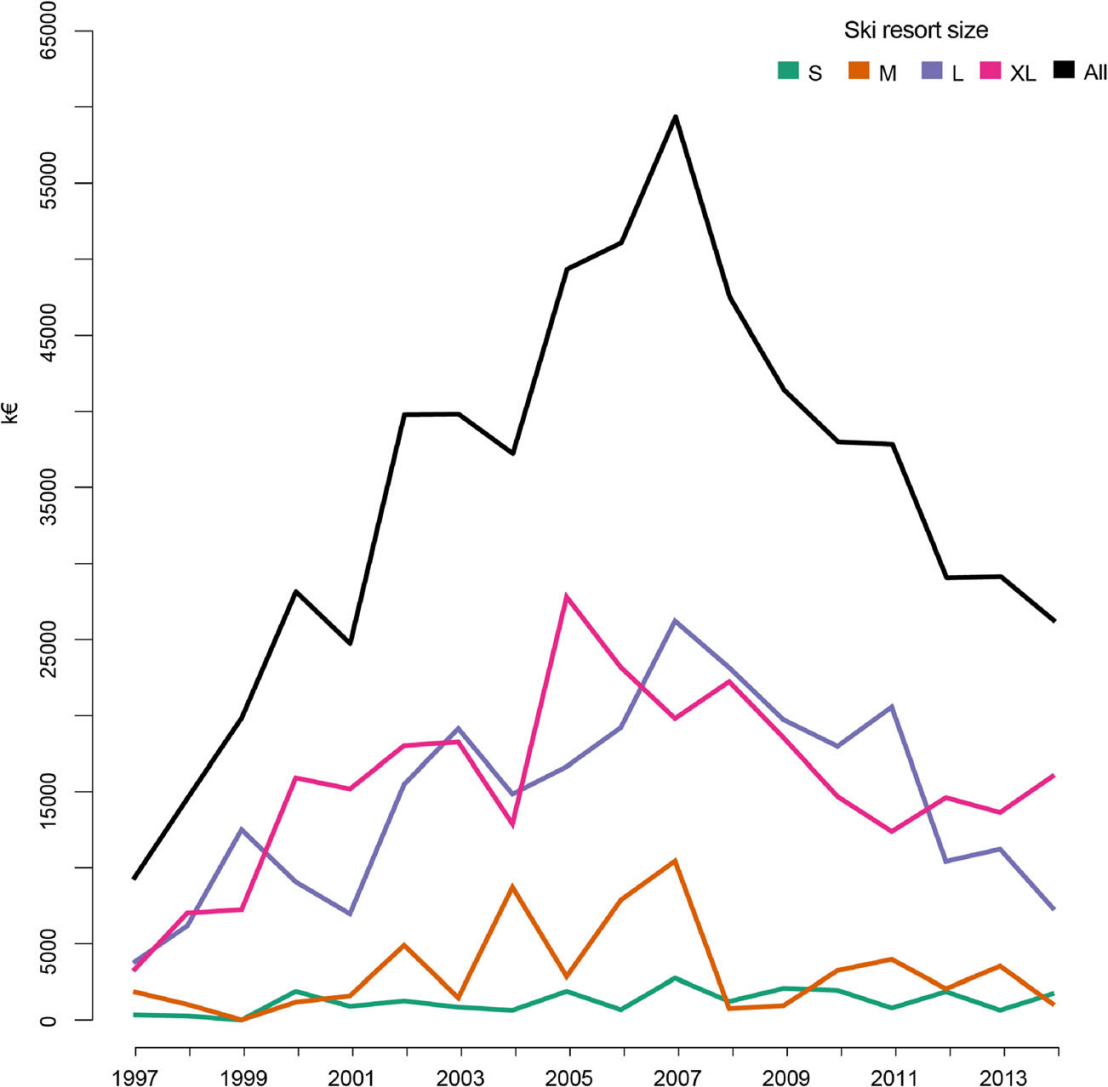
Evolutions of annual investments in snowmaking by ski resort size groups over the 1997–2014 period

Figure 2 provides the evolutions of investments in snowmaking by ski resort size over the 1997–2014 period. The overall snowmaking investment trend reached its peak in 2007. The 15 ski resorts from the XL group have invested as much as the 39 ski resorts from the L group.

**Fig. 3:**
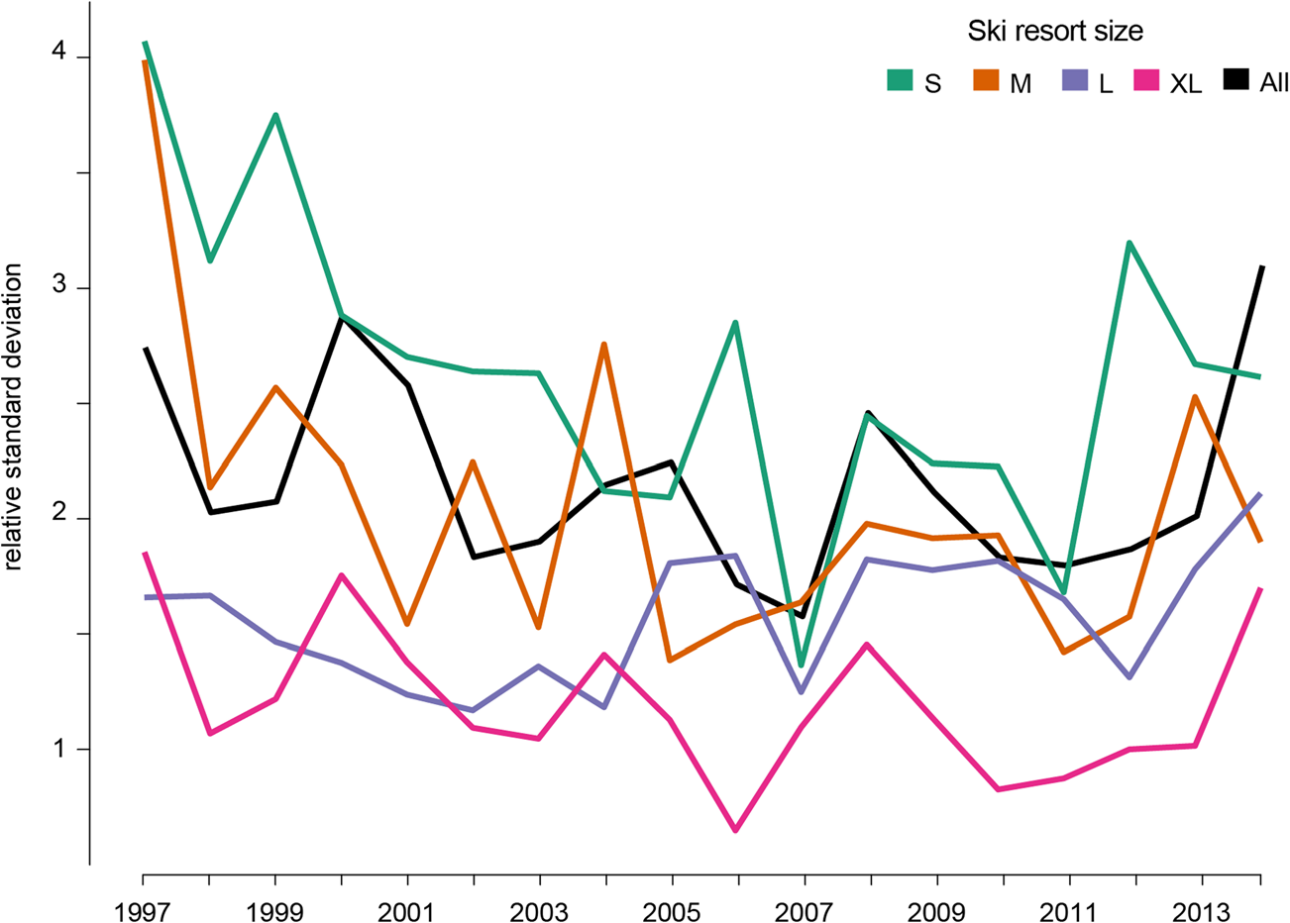
Within-year relative standard deviations of snowmaking investments, for various ski resort size categories (1997–2014)

Figure 3 captures the within-year relative standard deviations of snowmaking investments, for various ski resort size categories. The overall snowmaking investments dataset is highly heterogeneous, the relative standard deviation ranges from 1.6 to 3.1. Due to the spread of the distribution, snowmaking investment mean values are quite irrelevant. Figure 3 shows highest investment dispersion for small and medium ski resorts. The data indicate a more discontinuous investment behavior among these ski resorts, with several years without snowmaking investment. These first findings corroborate that the ski resort size is a key driver that has a strong influence on the snowmaking investment strategies.

**Fig. 4:**
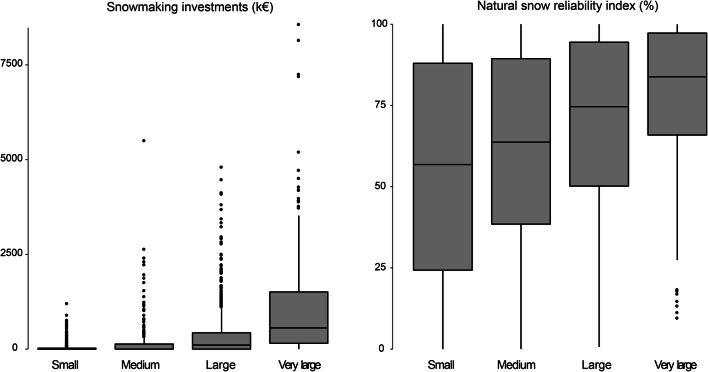
Overall interquartile ranges of snowmaking investments and natural snow reliability index regarding ski resort size

Table 5 displays descriptive statistics of natural snow reliability index and snowmaking investments regarding ski resort size group while Fig. 4 stresses overall interquartile ranges of both variables. Table 5 and Fig. 4 indicate a different snowmaking investment frequency regarding the ski resort size. Larger ski resorts invest higher amount and more frequently than smaller resorts. The snow reliability index is generally correlated to the ski resort size, which is due to the fact that larger resorts have mostly higher natural snow reliability ratios because of their higher elevation (François et al. 2014).

**Table 5:** Descriptive statistics of snowmaking investments and natural snow reliability index regarding ski resort size

Ski resort size	Variables	n	Mean	SD	RSD	Min.	25%	Median	75%	Max.
All	Index (%)	1800	65.3	30.0	0.45	0	42.8	71.6	93.0	100
	Inv (k€)	1800	350.341	775.035	2.21	0	0	39.618	341.838	8572.824
XL	Index (%)	270	77.7	22.1	0.28	9.6	65.9	83.8	97.3	100
	Inv (k€)	270	1051.95	1359.90	1.29	0	154.031	558.784	1508.13	8572.82
L	Index (%)	702	68.936	27.71	0.40	0.731	50.18	74.635	94.469	100
	Inv (k€)	702	375.35	663.91	1.77	0	0	107.947	431.11	4790.21
M	Index (%)	360	61.25	30.06	0.49	0.05	38.47	63.70	89.40	100
	Inv (k€)	360	165.15	464.75	2.81	0	0	0	134.37	5508
S	Index (%)	468	55.62	33.37	0.60	0	24.29	56.81	87.98	100
	Inv (k€)	468	50.51	137.79	2.73	0	0	0	24.28	1209

Snowmaking investments (*Inv*) are in constant price from 2014. *n* denotes the number of observation

**Fig. 5:**
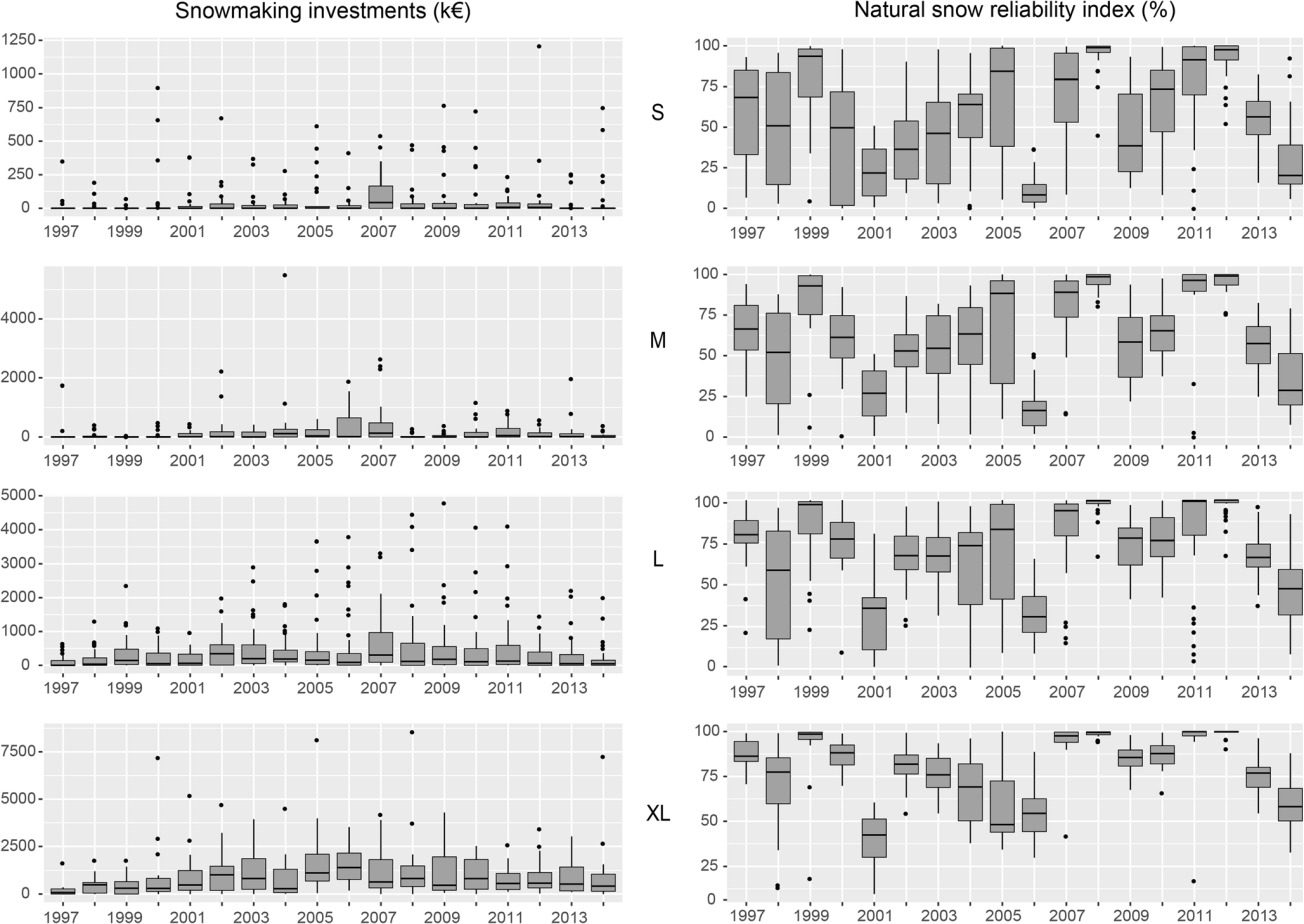
Interquartile ranges of snowmaking investments and snow reliability index according to ski resort size (1997–2014)

Figure 5 displays interquartile ranges of snowmaking investments and snow reliability index according to ski resorts size. It shows that investment boxplots feature a notable snowmaking investments increase in 2007 for small ski resorts (S) and to a lesser degree, for large resorts (L). The 2007 increase is less pronounced for medium ski resorts while it seems to happen in 2005 and 2006 for very large ski resorts (XL). Natural snow reliability index boxplots highlight the interannual variability of snow conditions. Table 8 in the Appendix shows within-ski resort deviations of natural snow reliability index and snowmaking investments (collection B). The relative standard deviation indicates a high dispersion of snowmaking investments.

To assess the potential size-related ski lift operator behavior and test our first assumption, we split our sample in two groups: on one side, large (L) and very large (XL) ski resorts constitute the larger ski resort sample (*n* = 972) while the second sample contains smaller (M and S) ski resorts (*n* = 828). Using more than 2 groups—i.e., one for each ski resort size group—would reduce the number of observations and decrease the robustness of the analysis.

### Empirical results

Tables 6 and 7 provide results of the 4 estimators used in our econometric analysis for larger (L and XL) and smaller (M and S) ski resorts.

**Table 6:** Impacts of prior year snow reliability on snowmaking investments on the larger ski resorts sample (L and XL)

Variable	Pooled_OLS	FE_FD	FE_WITHIN	RE_GLS
Snow reliability *(t −* 1*)*	− 0.368 (1.224)	− 0.913 (1.181)	− 1.465 (1.832)	− 1.217 (1.423)
Constant	615.338 *** (94.586)	11.047 (41.333)		677.005*** (123.664)
Number of observations	918	864	918	918
Number of ski resorts	54	54	54	54

*p < 0.1; **p < 0.05; ***p < 0.01. Estimations are based on clustered adjusted standard errors

**Table 7:** Impacts of prior year snow reliability on snowmaking investment on the smaller ski resorts sample (S and M)

Variable	Pooled_OLS	FE_FD	FE_WITHIN	RE_GLS
Snow reliability *(t − 1)*	− 1.148*** (0.369)	− 1.552*** (0.416)	− 1.240** (0.619)	− 1.300*** (0.384)
Constant	171.525*** (24.965)	1.513 (16.528)		180.597*** (29.966)
Number of observations	782	736	782	782
Number of ski resorts	46	46	46	46

*p < 0.1; **p < 0.05; ***p < 0.01. Estimations are based on clustered adjusted standard errors

The comparison between the 2 tables indicates here significant results for small and medium ski resorts sample (Table 7) rather than larger ski resorts sample (Table 6).

Concerning the larger ski resorts sample (XL and L) from Table 6, the relationship between the lagged snow reliability index and the snowmaking investments is not significant. None of the estimators shows significant results. Thus, we do not discuss further the results for larger ski resorts.

Table 7 shows that, regardless of the estimator for smaller ski resorts group, the snow reliability the year before has a significant impact on snowmaking investment. The coefficient sign is negative for all the estimators. The similarity of the estimated coefficients suggests that our estimations are not suffering from effects of unobserved heterogeneity.

We do not reject the null hypothesis (H_0_) with the Hausman test (*p* value = 0.9642). This indicates that both estimators are consistent but the random effects estimator (RE_GLS) is a more efficient option rather than within-transformation (FE_WITHIN). The Breusch-Pagan LM test is significant (*p* value < 0.01); it confirms that the pooled estimator is less appropriate because of the presence of panel effects (Greene 2019).

These results partially corroborate that a negative relationship with a time lag exist between a meteorological variable and the economic conduct of ski resorts. Poor snow conditions lead to an increasing snowmaking investment in the next year only for small and medium ski resorts. Such a significant relationship does not exist regarding large and very large ski resorts.

## Discussion

The aim of this study was to identify whether snowmaking investments in the French Alps resorts are affected by the ski resort size and the prior year natural snow conditions. We have used 4 estimators using a panel data set with 100 ski resorts in the French Alps, spanning several sizes and geographical settings. According to the results of our study, a snowmaking investment pattern seems to exist. Regardless of the resort size, over the past decades, ski lift operators dedicated around 20% of their investments for snowmaking. Snowmaking is an essential investment in this capital-intensive sector, for all ski resort sizes. The mean resort elevation does not seem to hold explanatory power in explaining changes in snowmaking investments apart from the lowest ski resorts. The snowmaking investment proportion is higher for the lowest resorts. Largest ski resorts invest higher amounts in snowmaking facilities. However, the frequency of occurrence of snowmaking investments differs substantially between larger and smaller ski resorts. Our results partly confirm the relevance of ski resort size to analyze snowmaking investments. This study also clearly shows that a negative short-term relationship exists between snowmaking investments and the prior year snow conditions for the small and medium ski resorts. There is no evidence that the same relationship exists for large and very large ski resorts.

We can interpret in different ways the absence of significant results for largest ski resorts sample. On the one hand, larger ski resorts are generally located at a higher elevation than smaller and thus are less sensitive to poor snow conditions (François et al. 2014). On the other hand, their ability to have snowmaking investment plans regardless of natural snow conditions can also explain the lack of significant results. Larger ski resorts can likely set up snowmaking investment strategies that go further than short-term reaction strategies as observed with smaller ski resorts. Snowmaking investment is an integral component of ski resort’s investment plans and steady snowmaking investments for many years may have also reduced the natural snow conditions sensitivity of larger ski resorts. This conduct has led larger ski resorts to be one the most equipped with snowmaking facilities.

Larger ski resorts might have motivations for snowmaking investments that are independent on the snow reliability of the previous year. Larger ski resorts find an advantage by setting a reinvestment strategy. Investments are necessary to remain efficient in a mature and competitive ski market. From an accounting perspective, investments give rise to depreciation and amortization which reduce company taxable income. However, the main finding that a relationship governs snowmaking investments based on prior-year snow conditions for small and medium ski resorts should be tempered. If a poor snow season can boost a snowmaking investment decision the year after, a succession of many extreme warm winters can lead ski lift operators to a critical financial position. Such a situation can also be damaging for the ski resort image with the risk of a permanent shift in demand to other ski resorts.

Our study suffers from both model and data used limitations. The natural snow reliability index is the only explanatory variable in our model; thus, it has not considered several factors (e.g., financial ratio, management model, risk perception of ski resort operators) that might influence investment ability and decision. Introducing other panel data would be appropriate to reinforce the specification of our model. Although the evolution of the ski lift power within a ski resort shows generally small changes over the time period considered, its evolution over the years for each ski resort could be better to analyze the ski resort size effect rather than the use of size groups.

A comparison between estimations based on different short-term periods could lead to assess whether snow conditions effects are stationary in time. In addition, the more ski resorts have invested, the less their sensitivity to natural snow conditions should be pronounced because snowmaking inherently reduces snow reliability hazard. This effect is not captured in our analysis, given that our snow reliability index is only based on natural snow conditions. Accounting for variations of snowmaking fractional coverage for individual ski resort is currently not possible in lack of sufficient data on snowmaking equipment rate. Our analysis of snowmaking investment amount could help fill in this gap in the future. The coefficient of the relationship may change over time. It can decrease if the ski resort operators reach a threshold for snowmaking facilities or face a decreasing accessibility to water supply. Changes in legal rules and public support can also have a noticeable influence. Overall, the gradual decrease over time of the intensity of the relationship could be investigated in further studies.

The use of a 1-year time lag in our model is a basic attempt to analyze operator behavior in response to meteorological conditions. Our econometric modeling only aims at assessing the past year’s influence on snowmaking investment. This simplification does not claim to convey all the complexity of the ski lift operator investment strategy. Ski lift operators probably also establish their snowmaking investment decisions on their feedback experience. Thus, the yearly time scale is possibly not sufficient to properly analyze snowmaking investment strategies. Depending on the financial capacities of ski resorts, these investments can be considered on a pluriannual term and some of them are mid-term planned. While some years are characterized with a high amount invested in structural facilities (e.g., water retention dams or snow production systems) they are followed by years with smaller investments such as extension of snowmaking facilities or machine replacement. The threshold effect might also exist in snowmaking development. For instance, a new dam or artificial lake has to be planned if an existing snowmaking system is not provided with enough water supplies. Snowmaking facility authorization processes take time and can also call for a multi-year planning strategy. Our modeling could be improved to capture a broader understanding of the snowmaking investment phenomenon. Past snowmaking investments as well as past snow events’ memories and shocks can influence current investments. Such a ski lift operator complex behavior can justify the use of a dynamic econometric modeling. To do so, the difference GMM or the system GMM (generalized method-of-moments) estimators as proposed by Arellano and Bond (1991) and Blundell and Bond (1998) could be employed. These estimators would fit with our panel data with few time periods (*t* = 18) and many individuals (*n* = 100). Although difference and system GMM are very popular including tourism industry studies (Falk and Tveteraas 2019; Töglhofer et al. 2011; Garín-Muñoz and Montero-Martín 2007), they remain complex to implement and can easily generate invalid estimates (Roodman 2009). We also must pay attention to the covered period (1997–2014) analyzed in our article. Although the relationship between poor snow seasons and the onset of use of snowmaking at the end of the 1980s and at the beginning of the 1990s is widely accepted, our panel data series do not cover this pioneer period. This era was a trial and error period for snowmaking development where technical and physical issues were the main concerns (SEATM 1989; Martin et al. 1990) rather than a precise economic assessment. It took several years before a systematic economic survey emerged with reliable data. Due to the lack of investment data, a quantitative approach seems hardly possible on that earlier period.

We underline the influence of meteorological conditions for small and medium ski resorts but although they are significant, they are not sufficient to explain ski resort use of snowmaking. We focus our study on a specific data, investments which are hardly available for other European ski resorts. To our knowledge, the analysis of snowmaking investment motivations has seldom been addressed because of the lack of data. There is no equivalent study about European ski resorts with the exception of Gonseth’s thesis (2008) using a sample of 87 Swiss ski resorts. The high specificity and the time span of our dataset make it nearly unique. Investment time series can be exploited in many different ways for vulnerability assessment, economic assessment as Falk and Vanat did (Falk and Vanat 2016) or to understand economic dynamics of ski resorts (Falk and Tveteraas 2019). However, potential results would be hardly comparable to other European ski resorts. Beyond the intrinsic limitations of our study, several elements indicate that snow reliability conditions are only one aspect to be considered in order to analyze ski resort investment strategies. The interest in public supports for the ski industry is often overlooked, although it is a widespread feature and has an influence on snowmaking investments. As a key socio-economic sector for mountainous regions, French ski resorts profit from public support (George-Marcelpoil and François 2012). The support from local authorities is not specific to France. For Switzerland, Gonseth (2008) provided a detailed explanation of both the different public stakeholders and public aids that can help ski resorts. He highlighted that public sector is deeply engaged in snowmaking investment support and he described a complex multi-scale system, with federal, cantonal, and local stakeholders. He also provided an overview of a specific law dedicated to support investments in mountainous areas. He laid down that the average share of public funds to supported snowmaking projects represented around 34% (Gonseth, p.40). Falk and Steiger (2018) also mentioned government support and public ownership in Austria. The question of public support to ski lift operator is often controversial in France. National authorities have stopped any direct support to ski resort development including snowmaking (George-Marcelpoil and François 2012). However, regional and local authorities are more inclined to support ski resorts. The French Alps encompass two administrative regions (NUTS 2): *Auvergne-Rhône-Alpes* (AuRA) and *Provence Alpes et Côte d’Azur* (Région Sud) *régions*. Both regions have set granting investment subsidies for ski resorts since 2014. *Département*, which is an administrative subdivision of regions (NUTS 3-level) with intrinsic political power, can also directly support snowmaking investments together with *régions*. Beside regional involvements, local authorities play a key role with financial as well as logistical support, even in the case where they do not directly own or manage a ski resort. Public management for ski resorts is not unusual, especially for small ones: according to the BD Stations, in the French Alps, 63 out of 139 ski resorts are publicly managed (45%), they only correspond to 15% of the total ski lift power. Out of 65 small ski resorts, 51 are managed publicly and the other 14 are mainly managed by non-profit organizations or publicly owned companies. Regarding publicly owned ski resorts, they likely fulfill other requirements than their profitability. Hence, two concerns appear: on one side an impact assessment of public support plans for ski resorts and snowmaking, on the other side a better understanding of local authorities as a key stakeholder in ski resort snowmaking investment strategy. The first is temporary while the second is organizational and both elements are not mutually exclusive. Beyond this political support of ski tourism through snowmaking investments, the socio-economic context in which snowmaking development occurred should not be overlooked. Snowmaking business as well as legal frameworks, ecological concerns (Paccard 2010), technical improvements (Campos Rodrigues et al. 2018) and ski tourism market evolution (Steiger and Mayer 2008), are all factors that have influenced the snowmaking development. As investments decision rests on multiple factors and snow conditions are only one of them, clearer indications about ski lift operator motivations to snowmaking are necessary to go beyond this quantitative analysis. A qualitative approach, with in-depth interviews and questionnaire-based surveys, could lead to a better analysis of ski lift operator behavior with respect to snowmaking investments and will form the basis of future studies.

## Appendix

**Table 8:** List of ski resorts from collection B with within-ski resort deviations of natural snow reliability index and snowmaking investment based on an 18-year period (1997–2014)

Ski resort	Size	Ski lift mean elevation (masl)	Ski lift power (km.pers/h)	Natural snow reliability index		Snowmaking investments	
				Annual mean (%)	Full period SD	Annual mean (k€)	Full period SD
Col Saint Jean	M	1885	2952	34	29	110	215
Stations de l’Ubaye	L	1910	5825	37	31	62	186
Pra-Loup	L	2058	6772	48	33	333	497
Val d’Allos	L	2040	8257	48	33	376	564
Station du Queyras	L	2067	6834	48	36	494	869
Ancelle	S	1511	1842	52	31	232	306
Stations du Champsaur	M	1692	3907	47	26	167	257
Les Orres	L	2029	6545	60	35	556	943
Montgenèvre	L	2156	8587	67	32	588	731
Orcières Merlette	L	2204	8297	76	28	296	481
Pelvoux-Vallouise	S	1615	1391	54	31	79	94
Puy Saint Vincent	L	2043	5734	78	29	212	346
Réallon	S	1792	1408	48	36	27	73
Risoul	L	2190	6734	68	37	432	976
Serre Chevalier	XL	1999	26,571	73	27	1197	1264
Massif du Dévoluy	L	1834	7068	59	29	522	798
Vars	L	2144	9073	59	36	637	991
Chazelet-Villar d’Arene	S	1898	1088	70	29	29	109
Stations du Mercantour	XL	2031	17,669	57	32	1908	2200
Roubion Les Buisses	S	1629	728	28	31	19	82
Valberg-Beuil	M	1656	4849	36	36	763	1374
Col Du Rousset	S	1433	1297	58	34	0	2
Lus-la-Jarjatte	S	1357	385	25	28	0	2
Autrans	S	1420	1535	42	32	9	16
Chamrousse	L	1883	7078	89	18	138	166
Le Collet d’Allevard	M	1715	2897	77	25	45	104
Gresse-en-Vercors	S	1410	1257	47	32	34	67
Saint Pierre de Chartreuse	M	1318	2958	52	33	2	8
L’Alpe d’Huez	XL	2129	18,232	73	24	1498	2380
L’Alpe du Grand Serre	M	1716	3225	58	31	64	269
Les Deux Alpes	XL	2311	23,796	83	18	361	347
Saint Hilaire du Touvet	S	1075	517	27	24	16	63
Les Sept Laux	L	1786	10,881	78	23	423	1065
Oz-Vaujany	L	1853	8072	65	26	257	580
Lans-en-Vercors	S	1523	1880	60	35	13	22
Méaudre	S	1265	1645	32	26	29	92
Col de Marcieu	S	1194	221	41	35	26	98
Villard-de-Lans	L	1575	9644	61	30	522	757
Albiez-Montrond	M	1725	2708	73	26	137	365
Arêches-Beaufort	M	1652	4247	74	20	122	253
Aussois	M	2096	3055	55	24	288	509
Bonneval-sur-Arc	S	2339	2024	72	31	30	41
Crest Voland	M	1411	3472	58	34	202	312
Val d’Arly	L	1506	8345	71	25	300	339
Savoie Grand Revard	S	1407	1287	70	32	1	3
La Norma	M	2018	4032	54	29	262	397
La Plagne	XL	2061	35,044	74	21	1156	1023
La Rosiere	L	2033	6969	70	26	276	798
La Toussuire	L	1940	6148	72	24	322	536
Le Corbier	L	1865	6363	71	22	195	344
Les Arcs	XL	2019	31,699	76	23	1599	2063
Les Karellis	M	2043	4986	84	17	118	197
Les Menuires	XL	2189	22,331	79	20	834	692
Les Saisies	L	1739	8433	74	23	477	709
Méribel	XL	1878	15,767	64	23	948	869
Pralognan	M	1813	3505	52	28	82	104
Saint-François-Longchamp	L	1904	6405	62	27	105	225
Saint-Sorlin d’Arves	L	2028	7746	75	22	201	363
Tignes	XL	2443	25,814	88	17	677	932
Val Cenis	L	1927	13,212	50	29	361	357
Val d’Isère	XL	2381	24,371	87	18	1333	833
Val Fréjus	M	2145	3773	57	30	159	443
Valloire	L	1951	9631	77	20	666	1121
Valmeinier	L	2019	7718	80	19	481	696
Valmorel	L	1762	11,005	64	26	358	484
Val Thorens	XL	2501	19,844	91	14	1469	1641
Orelle	L	2294	5217	61	10	62	223
Bessans	S	1849	185	32	32	66	186
Sainte-Foy Tarentaise	S	2067	2436	74	25	89	193
Courchevel	XL	2094	39,787	79	20	1111	1184
Aillon Le Jeune-Margeriaz	M	1431	3594	64	26	9	30
Abondance	S	1375	1205	65	26	73	180
Avoriaz-Morzine	XL	1789	18,826	81	15	544	704
Bellevaux Hirmentaz	S	1362	2115	65	29	163	193
Bernex	S	1399	2372	68	25	118	228
Chamonix	XL	1939	27,378	82	15	524	604
La Chapelle d’Abondance	M	1381	3156	68	27	154	458
Chatel	L	1641	13,959	79	20	740	1164
Combloux	M	1541	4753	82	20	202	617
Les Contamines-Hauteluce	L	1786	10,409	76	20	427	470
Flaine	L	1987	13,466	93	9	545	789
Le Grand Bornand	L	1516	11,400	76	22	733	841
Habere Poche	S	1200	1454	51	29	19	75
La Clusaz	L	1633	13,826	82	18	641	847
Les Brasses	M	1249	2617	54	29	201	327
Les Carroz d’Araches	L	1562	7348	70	25	272	517
Les Gets	L	1502	10,489	72	26	472	527
Les Houches-Saint-Gervais	L	1536	5872	77	22	53	118
Manigod Croix Fry	S	1579	2088	86	22	111	216
Megève	XL	1562	15,132	80	21	619	946
Mont-Saxonnex	S	1346	828	77	25	8	17
Morzine Pleney Nyon	L	1468	9204	71	24	472	564
Plaine-Joux	S	1508	749	60	31	30	111
Praz-De-Lys-Sommand	L	1575	5099	78	24	21	72
Saint Gervais Bettex	L	1552	7293	77	19	441	564
Espace Roc d’Enfer	M	1425	3100	62	27	145	544
Morillon-Samoens-Sixt	L	1446	12,159	68	20	170	246
Thollon-Les-Memises	S	1538	2468	81	22	57	134
Saint Nicolas de Véroce	M	1751	3657	84	17	74	114
Sallanches-Cordon	S	1315	1005	61	29	35	96

## Footnotes

1 *Montagne Leaders* is very well known in the ski tourism industry and it is a key stakeholder that contributes to the organization of the national trade fair for mountain areas (*Mountain Planet*, formerly *Salon d’Aménagement de la Montagne*)

2 Discussion with Mickael Frottier (Editor-in-chief of *Montagne Leaders*).
